# Sexual and Reproductive Health Needs Assessment & Interventions in a Female Psychiatric Intensive Care Unit

**DOI:** 10.1192/j.eurpsy.2022.2088

**Published:** 2022-09-01

**Authors:** E. Covshoff, L. Blake, E. Rose, A. Bolade, R. Rathouse, A. Wilson, A. Cotterell, R. Pittrof, F. Sethi

**Affiliations:** 1 Guy’s and St Thomas NHS Foundation Trust, Sexual And Reproductive Health, London, United Kingdom; 2 South London and Maudsley Foundation Trust, Maudsley Hospital, AZ, United Kingdom; 3 Central and North West London NHS Foundation Trust, Perinatal Mental Health Service, London, United Kingdom; 4 South London and Maudsley NHS Foundation Trust, Eileen Skellern, London, United Kingdom; 5 Dorset HealthCare University NHS Foundation Trust, Executive Medical Director, Poole, United Kingdom

**Keywords:** Sexual health, mental health, psychiatric intensive care, women’s health

## Abstract

**Introduction:**

This quality improvement project was a collaboration between an adult, inpatient female psychiatric intensive care unit (PICU) in South London and the Sexual and Reproductive Health Rights, Inclusion and Empowerment (SHRINE) programme. SHRINE is a London-based programme delivering SRH care to any individual with serious mental illness, substance misuse and/or learning disability.

**Objectives:**

The primary aim of this quality improvement project was to assess patients’ sexual and reproductive (SRH) needs, and the acceptability of providing SRH assessments in a female PICU setting. Secondary aims were to explore the barriers to access and the feasibility of providing SRH assessments and SHRINE interventions in the PICU.

**Methods:**

A bi-monthly SRH in-reach clinic and a nurse led SRH referral pathway were implemented on the PICU over a seven-month period. Within a quality improvement framework, a staff training needs assessment was performed, training delivered, a protocol developed, staff attitudes explored, and patient and carer engagement sought.

**Results:**

30% of women were identified as having unmet SRH needs and proceeded to a specialist appointment, representing a 2.5-fold increase in unmet need detection. 42% of women were assessed, representing a 3.5-fold increase in uptake. 21% of women initiated SRH interventions of which 14% had all their SRH needs met.

**Conclusions:**

Results identified SRH needs for PICU admissions are greater than realised. Staff highlighted the acceptability and importance of SRH care, if interventions are appropriately timed and the patient’s individual risk profile considered. Providing a nurse-led referral pathway for an SRH in-reach clinic is acceptable, feasible and beneficial for PICU patients.

**Disclosure:**

No significant relationships.

